# *NUDT15* Polymorphism Confer Increased Susceptibility to Thiopurine-Induced Leukopenia in Patients With Autoimmune Hepatitis and Related Cirrhosis

**DOI:** 10.3389/fphar.2019.00346

**Published:** 2019-04-09

**Authors:** Xiaoli Fan, Dandan Yin, Ruoting Men, Heng Xu, Li Yang

**Affiliations:** ^1^Department of Gastroenterology and Hepatology, West China Hospital, Sichuan University, Chengdu, China; ^2^Department of Laboratory Medicine, National Key Laboratory of Biotherapy/Collaborative Innovation Center for Biotherapy and Precision Medicine Key Laboratory of Sichuan Province, West China Hospital, Sichuan University, Chengdu, China

**Keywords:** azathioprine, leukopenia, *NUDT15*, autoimmune hepatitis, cirrhosis

## Abstract

The aim of this study was to investigate the influence of *NUDT15 R139C* and thiopurine S-methyltransferase (*TPMT*) on azathioprine (AZA) induced leukopenia in patients with autoimmune hepatitis (AIH) and related cirrhosis. A total of 149 Chinese AIH patients with a history of AZA treatment were retrospectively evaluated. The clinical and epidemiological characteristics of the patients were obtained from an electronic database and reviewed. *NUDT15* (rs116855232) and *TPMT^∗^3C* (rs1142345) SNPs were genotyped using a PCR method. Twelve patients developed leukopenia, and this adverse drug reaction was significantly associated with the T risk allele in *NUDT15* [*P* < 0.00001, odds ratio = 20.41; 95% confidence interval (CI) (7.84, 53.13)], with the sensitivity and specificity of 91.67 and 89.05%, respectively. The median maintenance dosages for patients with the rs116855232 CC and CT genotypes were 1.23 (0.95, 1.53) mg ⋅ kg^−1^ ⋅ d^−1^ and 0.96 (0.83, 1.19) mg ⋅ kg^−1^ ⋅ d^−1^, respectively (*P* = 0.028). In contrast, no significant association was observed for *TPMT^∗^3C* genotypes. Notably, subgroup analysis of the 13 patients with leukopenia before therapy, these white blood cell (WBC) counts did not show further reduction after AZA treatment and maintenance dosage was 1.13 (0.94, 1.60) mg ⋅ kg^−1^ ⋅ d^−1^. Therefore, *NUDT15* polymorphism is significantly associated with thiopurine-induced leukopenia in Chinese patients with AIH and related cirrhosis. Adjusting the AZA dosage should be considered in patients according to the *NUDT15 R139C* genotypes.

## Introduction

Autoimmune hepatitis (AIH) is a chronic self-perpetuating inflammatory liver disease characterized by increased serum aminotransferase levels and autoantibodies, elevated serum immunoglobulin G (IgG) concentration, lymphoplasmacytic infiltration of the portal tract, and interface hepatitis. Misdiagnosis or treatment failure can lead to liver cirrhosis, liver cancer, liver transplantation and rapid death (European Association for the Study of the Liver, 2015; Manns et al., 2015; Montano-Loza and Czaja, 2015). The first-line treatment for AIH involves predniso(lo)ne as an initial therapy, followed by the addition of azathioprine (AZA) after 2 weeks. The initial dosage of AZA should be 50 mg ⋅ d^−1^, which can be increased based on the toxicity and response up to the maintenance dose of 1–2 mg ⋅ kg^−1^. However, most recent studies implied that neither Thiopurine S-methyltransferase (*TPMT*) heterozygosity nor 6-mercaptopurine (6-MP) metabolite levels were reliable predictors of AZA efficacy or toxicity in patients with AIH and related cirrhosis, and cytopenia is frequently encountered due to advanced fibrosis (Czaja and Carpenter, 2006; Heneghan et al., 2006; Hindorf et al., 2010).

Progressive cytopenia, white blood cell (WBC) counts below 3 × 10^9^/L, or platelet counts below 50 × 10^9^/L are severe hematological side effects that justify the discontinuation of the AZA treatment (Czaja and Carpenter, 2006). Leukopenia, which is one of the most common and life-threatening adverse effects of AZA treatment, poses a substantial challenge to clinical practice and treatment outcomes. TPMT is a key enzyme involved in AZA metabolism. Deficiency or low activity of TPMT may generate high concentrations of the active metabolite 6-thioguanine nucleotide (6-TGN), which accounts for the therapeutic effects of AZA but is also associated with bone marrow toxicity (Manns et al., 2015). The gene encoding the TPMT enzyme is controlled by germline polymorphisms that lead to varied levels of enzyme activity among individuals. The risk allele frequency of rs1142345 is high in Caucasians (4%), Latino (4.8%), and African (5.4%) populations but low in East Asian (1.3%) populations^1^. Paradoxically, thiopurine-induced leukopenia is more common in East Asian individuals, and many patients who develop leukopenia after receiving AZA treatment cannot be reliably identified by genotyping or TPMT activity measurements because these patients have normal or near normal TPMT activity (Connell et al., 1993; Takatsu et al., 2009; Kim et al., 2010; Fangbin et al., 2012). This suggests that the value of TMPT in predicting thiopurine-induced leukopenia in East Asians is limited (Takatsu et al., 2009; Fangbin et al., 2012) and that unknown factors could be responsible for the inter-individual variation in thiopurine sensitivity across ethnicities (Ban et al., 2008; Fangbin et al., 2012; Zhu and Cao, 2012; Lee et al., 2015).

A genome-wide association study found that a c.415 C-to-T transition (rs116855232) variant of the nucleoside diphosphate-linked moiety X motif 15 (*NUDT15*) gene results in a p.Arg139Cys substitution and is strongly associated with thiopurine-induced myelosuppression and thiopurine tolerance dose with underlying race-specific genetic polymorphisms; this result has been replicated by multiple independent studies (Yang et al., 2014; Tanaka et al., 2015; Zhu et al., 2016). We previously conducted a meta-analysis to investigate the impact of rs116855232 on thiopurine-induced myelotoxicity susceptibility and thiopurine intolerance dose. In this study, the rs116855232 variant allele resulted in a 7.86-fold (*P* < 0.00001, 95% confidence interval (CI): 6.13–10.08) increased risk of developing leukopenia with a high specificity (91.74%) and sensitivity (43.19%) and a lower thiopurine intolerance dose (*P* < 0.00001) (Yin et al., 2017). However, many previous studies focused mostly on acute lymphoblastic leukemia (ALL) (Yang et al., 2014), inflammatory bowel diseases (IBD) (Lee et al., 2016; Zhu et al., 2016), and other autoimmune diseases (Kim et al., 2017; Fei et al., 2018; Yan et al., 2018). No previous studies have focused on AIH, with the exception of our previous report on a case of AZA-induced lethal myelosuppression in a patient with AIH variant syndrome and the *NUDT15*^risk/risk^ *TPMT*^wt/risk^ genotype (Yang et al., 2017). As the patient group with AIH and related cirrhosis needs special attention, we conducted this retrospective study to explore the effects of *NUDT15* (rs116855232) and *TPMT* ^∗^3C (rs1142345) SNPs on AZA-induced leukopenia in Chinese patients. Additionally, we sought to confirm the impact of *NUDT15* heterozygosity on thiopurine-induced leukopenia and the tolerance dose to guide the clinical usage of thiopurines in this patient cohort.

## Materials and Methods

### Patients and Treatment

West China Hospital is a 4950-bed tertiary teaching hospital affiliated with Sichuan University; it has a liver transplant unit (Liver Transplantation Center, West China Hospital) and is the leading medical center in West areas of China. In total, 149 consecutive patients with AIH or AIH- primary biliary cholangitis (PBC) variant syndrome who prescribed AZA for more than 3 months were recruited at the West China Hospital between October 2013 and October 2017. Individuals were included if they met the criteria for definite or probable AIH based on the revised criteria published by the International AIH Group in 1999; AIH – PBC variant syndrome was diagnosed according to the Paris criteria (Chazouilleres et al., 1998). According to the recent EASL guidelines, the first line of treatment is prednisone(lo)ne followed by the addition of AZA after 2 weeks or when the bilirubin levels fall below 6 mg/dl (100 μmol/L). The AZA dose is increased based on the toxicity and response until the maintenance dose of 1–2 mg/kg is reached. Immunosuppressive agents in combination of ursodesoxycholic acid (UDCA) are often recommended for patients with AIH – PBC variant syndrome in clinical practice (Hirschfield et al., 2017).

Complete blood counts were performed weekly during the 1st month of AZA treatment, biweekly in the following 2 months, and monthly thereafter. The researchers performed follow-up via telephone and internet communication.

The primary endpoint was leukopenia, which is defined as a WBC count less than 3 × 10^9^/L. For patients whose WBC counts were less than 3 × 10^9^/L before therapy, leukopenia was deemed progressive deterioration after treatment. The secondary endpoint was drug withdrawal for persistent or intolerant leukopenia. The dose was reduced in patients who developed leukopenia. If any laboratory abnormality did not subside, AZA was discontinued. Decisions regarding discontinuation or dose adjustment were made by the physician responsible for the personalized therapy.

This study was approved by the Ethics Committee of West China Hospital. Written informed consent was obtained from all enrolled patients.

### Genetic Analyses and Azathioprine Metabolites

Total genomic DNA was extracted from peripheral blood using the Blood DNA Mini Kit (FOREGENE) according to the manufacturer’s instructions. Genotypes of rs116855232 and rs1142345 were determined by polymerase chain reaction (PCR) and Sanger sequencing. The sequences of the forward and reverse primers for rs116855232 were AAGCAAATGCAAAGCATCAC and GGCTGAAAGAGTGGGGGATA, and the sequences of the forward and reverse primers for rs1142345 were ATAGGCATAATCTTTCAC and CAGCCAATTTTGAGTATTT. PCR amplification for rs116855232 starts with an initial denaturation at 94°C for 5 min, followed by 35 cycles of 95°C for 30 s, 65°C for 30 s and 72°C for 60 s. Final extension was performed at 72°C for 10 min, and samples were stored at 12°C. The PCR reaction for rs1142345 starts with an initial denaturation at 94°C for 5 min, followed by 35 cycles of 95°C for 30 s, 54°C for 30 s and 72°C for 60 s. Final extension was performed at 72°C for 10 min, and samples were stored at 12°C. Measurement of AZA metabolites uses high performance liquid chromatography and results are reported in pmol/8^∗^10^8^ RBCs, as previously described (Dervieux and Boulieu, 1998). All procedures were conducted in the Department of Laboratory Medicine of West China Hospital, which was certified by the College of American Pathologists (CAP).

### Definition of Remission

The treatment responses were classified according to biochemical remission. AIH biochemical remission was defined as the normalization of transaminases and IgG, as determined using existing guidelines.

### Statistical Analysis

Continuous data are presented as medians and quartiles, and categorical data are expressed as percentages. The Mann–Whitney *U*-test was performed to compare continuous variables. Chi-square tests or Fisher’s exact tests were performed to analyze differences in categorical variables between two independent groups as appropriate. The odds ratios (OR) and 95% CIs associated with rs116855232 were determined by performing logistic regression analyses assuming log-additive, dominant and allele genetic models of inheritance using Review Manager 5.3 software. A two-side *P*-value less than 0.05 were considered statistically significant. Statistical analysis was performed using the SPSS software package (SPSS version 22.0 for Windows, IBM Corp., Armonk, NY, United States).

## Results

### Patient Characteristics

In total, 149 consecutive patients with AIH were included in this study from October 2013 to October 2017. The female-to-male ratio was 6.95:1. Of these patients, 70 patients were diagnosed with variant AIH-PBC syndrome. Eighty-one patients were diagnosed with cirrhosis at the initiation of AZA therapy. After AZA treatment, leukopenia was observed in 12/149 patients (8.1%), 3 of whom also had transient gastro-intestinal reactions, and 2 of whom developed mild hair loss. Other side effects included pancreatitis (*n* = 1), bone aches (*n* = 1), and herpes zoster (*n* = 1). For detailed patient characteristics, see Table 1. No significant differences were observed in age, sex, indices of liver function, disease severity or baseline WBC counts at diagnosis between the individuals with or without thiopurine-induced leukopenia (*P* > 0.05) (Table 1).

**Table 1:** Baseline characteristics of the 149 patients included according to azathioprine (AZA) induced leukopenia.

Clinical features	With leukopenia (*N* = 12)	Without leukopenia (*N* = 137)	*P*
Age, y	49.0(46.0, 63.5)	52.0(44.0, 58.5)	0.997
Female	10(83.3%)	119(86.9%)	>0.999
TBil, umol/L	29.8(19.4, 55.0)	29.6(14.5, 52.2)	0.807
ALT, IU/L	142.0(42.5, 181.3)	101.0(48.0, 189.5)	0.976
AST, IU/L	137.0(48.3, 208.0)	98.0(44.0, 180.0)	0.577
ALB,g/L	39.3(33.5, 40.9)	39.6(35.1, 42.8)	0.463
GLB,g/L	42.0(34.6, 47.2)	37.0(31.9, 42.6)	0.083
ALP, IU/L	139.0(93.5, 230.5)	154.9(98.5, 267.5)	0.867
GGT, IU/L	124.5(37.0, 231.8)	137.0(66.0, 301.5)	0.330
IGG, IU/L	24.0(17.1, 29.6)	18.3(15.3, 25.3)	0.244
IGM, IU/L	2.5(1.8, 3.6)	2.3(1.5, 3.7)	0.861
Cirrhosis (*n*, %)	8/12(66.7%)	73/137(53.3%)	0.372
Splenomegaly (*n*, %)	6(50.0%)	44(32.1%)	0.348
WBC at diagnosis, ×10^9^/L	5.1(4.0, 5.9)	5.0(3.9, 6.4)	0.947
AIH/AIH-PBC	6/6	73/64	0.827
rs116855232 CC/CT/TT	1/9/2	122/15/0	<0.001
rs1142345 TT/TC/CC	11/1/0	134/2/1	0.283

Of the 149 analyzed subjects, 123 subjects displayed wild-type *NUDT15* (CC; 82.6%). Twenty-four subjects were *NUDT15* heterozygotes (CT; 16.1%), and two subjects were homozygotes (TT; 1.3%), respectively. The variant allelic frequency of rs116855232 was 9.4%, while *TPMT^∗^3C* alleles were only observed in four subjects including 3 patients who were heterozygotes (TC) and 1 patient who was homozygous (CC). Significant differences were observed in the rs116855232 genotypes between the individuals with or without leukopenia (*P* < 0.05).

### Association Between the rs116855232 Genotypes and Leukopenia

For detailed patient characteristics about association between the rs116855232 genotypes and leukopenia according to *NUDT15* genotypes, see Table 2.

**Table 2:** Combined analysis patients according to *NUDT15* genotype.

	CC	CT	TT
Total patients	123	24	2
Female (*n*, %)	107(87.0%)	21(87.5%)	1(50.0%)
Age	52.0(45.0, 58.0)	48.0(40.5, 62.8)	46 and 68, respectively
Observation duration (weeks)	12.0(5.0, 16.0)	16.5 (8.3, 20.5)	5 and 7 months, respectively
AIH/Overlap	67/56	11/13	1/1

*Data were compared between patients of NUDT15 genotype.*

**Table 3:** Association between *NUDT15* SNP (rs116855232) and risk of leukopenia, AZA maintenance dosage, AZA interruption.

	CT (*n* = 24)	CC (*n* = 123)	TT (*n* = 2)	Dominant model OR (95% CI) *P*-value	Recessive model OR (95% CI) *P*-value	Allele model OR (95% CI) *P*-value
**Leukopenia**						
Yes	9 (37.5%)	1 (0.8%)	2 (100.0%)	89.47 (10.78, 742.53) *P* < 0.0001	65.48 (2.95, 1454.21) *P* = 0.008	20.41 (7.84, 53.13) *P* < 0.00001
No	15 (62.5%)	122 (99.2%)	0 (0%)			
**Leukopenia during first 8 weeks**						
Yes	9 (37.5%)	1 (0.8%)	2 (100.0%)	89.47 (10.78, 742.53) *P* < 0.0001	65.48 (2.95, 1454.21) *P* = 0.008	20.41 (7.84, 53.13) *P* < 0.00001
No	15 (62.5%)	122 1(99.2%)	0 (0%)			
**Maintenance dosage**						
≤1.0mg ⋅ kg^−1^ ⋅ d^−1^	8/17 (47.1%)	23/75 (30.6%)	1/1 (100.0%)	2.26 (0.79, 6.44)	5.86 (0.23, 147.96)	2.33 (0.89, 6.06)
>1.0mg ⋅ kg^−1^ ⋅ d^−1^	9/17 (52.9%)	52/75 (69.3%)	0/1 (0%)	*P* = 0.13	*P* = 0.28	*P* = 0.08
**Therapy interruption**						
Yes	2/24 (8.3%)	6/123 (4.9%)	1/2 (50.0%)	2.54 (0.59, 10.91)	17.38 (0.99, 303.99)	3.05 (0.93, 9.99)
No	22/24 (91.7%)	117/123 (95.1%)	1/2 (50.0%)	*P* = 0.21	*P* = 0.05	*P* = 0.07

*SNP indicates single nucleotide polymorphism.*

Therapy-induced leukopenia was observed in 9/24 (37.5%) patients with the CT allele and 2/2 (100%) patients with the TT allele. However, only one patient with wild-type homozygote allele experienced leukopenia. All cases of leukopenia developed within the first 60 days following therapy initiation or dose increase. Three of the nine (33.3%) patients developed leukopenia during remission induction, while the other 6 (66.7%) developed leukopenia during the maintenance therapy. One patient with the TT genotype experienced lethal levels of myelosuppression in the first 4 weeks of remission induction with a 1.09 mg ⋅ kg^−1^ ⋅ d^−1^ prescription and reached the diagnostic criteria for grade 4 myelosuppression; this patient also carried a *TPMT*^wt/risk^ genotype. Although the AZA treatment was immediately halted and human granulocyte colony-stimulating factor were prescribed to increase blood cell counts, the patient suffered a life-threatening progressive decrease in blood cell number. Subsequently, a second-line immunosuppressant (mycophenolate mofetil) was recommended after recovery from the myelosuppression. The other patient with the TT genotype was reduced to a 0.2 mg ⋅ kg^−1^ ⋅ d^−1^ prescription after the WBC count from 5.29 × 10^9^/L dropped to 1.94 × 10^9^/L at the 0.9 mg ⋅ kg^−1^ ⋅ d^−1^ prescription; also, WBC counts gradually increased to a satisfactory value (3–4.5 × 10^9^/L) after the dosage adjustment.

When evaluated by rs116855232 genotype, leukopenia was significantly associated with the T allele [*P* < 0.00001, odds ratio = 20.41; 95% CI (7.84, 53.13)], with the sensitivity and specificity of 91.67 and 89.05%, respectively (Table 3). The NUDT15 variant allele had a high predictability for leukopenia (42.3%, 11/26).

### Association Between the rs116855232 Genotype, 6-TGN Levels and Maintenance Dose

**FIGURE 1:**
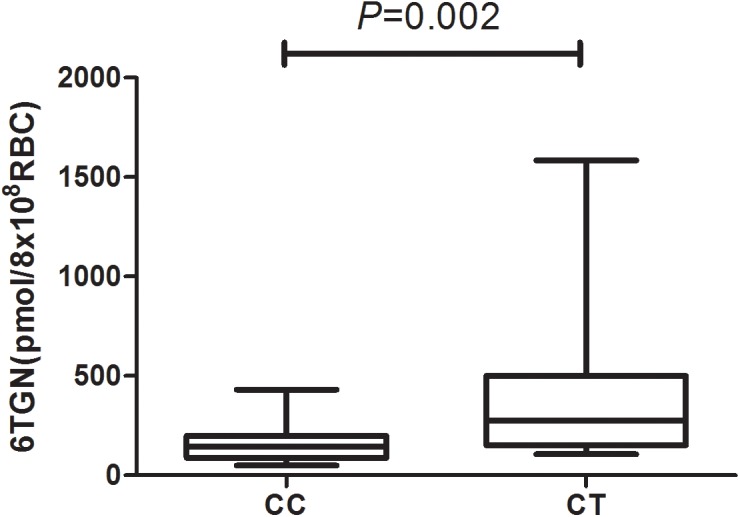
6-Thioguanine nucleotide concentrations for patients with rs116855232 CC and CT genotypes were 145.3(87.4, 199.0) pmol/8 ^∗^10^8^ RBC and 276.6(150.9, 501.0) pmol/8^∗^10^8^ RBC, respectively (*P* = 0.002).

6-Thioguanine nucleotide concentrations were significantly higher in the patients of *NUDT15* CT genotype [*P* = 0.002, 276.6(150.9, 501.0) vs. 145.3(87.4, 199.0) pmol/8^∗^10^8^ RBC] (Figure 1 and Supplementary Table S1).

**FIGURE 2:**
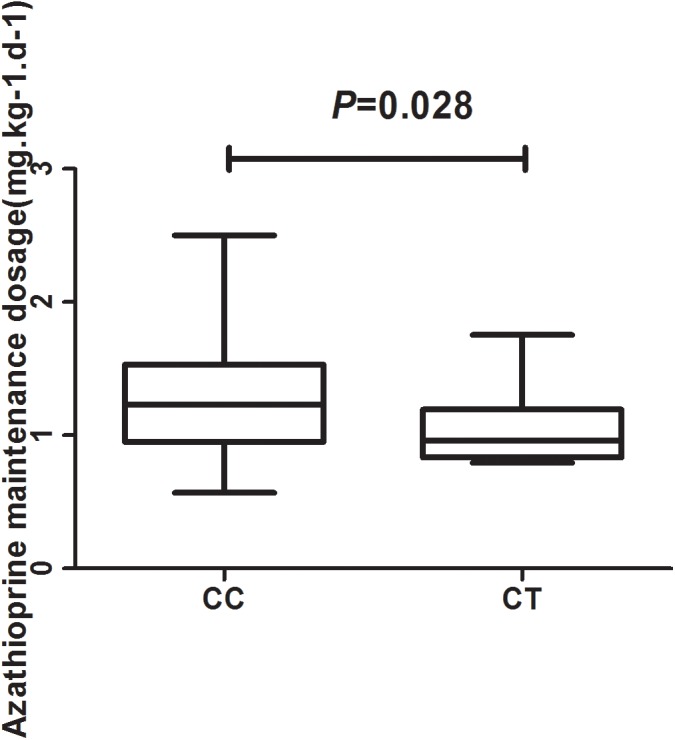
The median maintenance dosages for patients with rs116855232 CC and CT genotypes were 1.23 (0.95, 1.53) mg ⋅ kg^−1^ ⋅ d^−1^ and 0.96 (0.83, 1.19) mg ⋅ kg^−1^ ⋅ d^−1^, respectively (*P* = 0.028).

**FIGURE 3:**
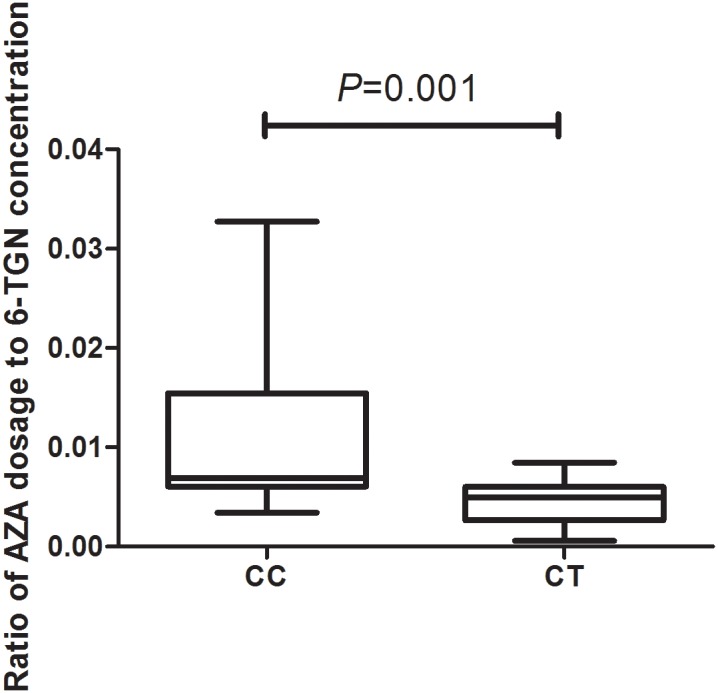
The median ratio of thiopurine dose to 6-TGN concentration for the patients with rs116855232 CC and CT genotypes were 0.007(0.006, 0.015) and 0.005(0.003, 0.006; *P* = 0.001).

In total, 93 patients have achieved biochemical remission and began maintenance therapy as of February 2018. The AZA dose did not significantly differ between CC and CT genotypes (*P* = 0.22) when evaluated with a clinical therapeutic dose of 1.0 mg ⋅ kg^−1^ ⋅ d^−1^. However, the median maintenance dosages for patients with the *NUDT15* CC and CT genotypes were 1.23 (0.95, 1.53) mg ⋅ kg^−1^ ⋅ d^−1^ and 0.96 (0.83, 1.19) mg ⋅ kg^−1^ ⋅ d^−1^, respectively (*P* = 0.028) (Figure 2). The ratio of AZA dose to 6-TGN concentration for the patients was compared between *NUDT15* CC and CT genotypes and the results showed that patients with *NUDT15* CT genotypes had much lower values (*P* = 0.013) (Figure 3). The maintenance dose associated with the *NUDT15* TT genotype did not reach statistical significance, as only one patient with the *NUDT15* TT genotype continued AZA therapy (0.2 mg ⋅ kg^−1^ ⋅ d^−1^).

### Association of 6-TGN Levels With Leukopenia

The 6-TGN concentration of 61 patients ranged from 49.74 to 1583.38 pmol/8^∗^10^8^ RBC, and median 6-TGN concentration was 149.1 pmol/8^∗^10^8^ RBC. The associations of concentrations of 6-TGN with different groups were analyzed in Supplementary Table S1. Overall, the level of 6-TGN was not significantly different between patients with leukopenia or without leukopenia (*P* = 0.149), and no significant difference was observed between two groups, regarding to gender, AZA maintenance dosage and severity of liver disease (*P* = 0.379, 0.637, and 0.757, respectively).

### Subgroup Analysis of Patients With Previous Leukopenia

In the 13 patients with leukopenia before therapy, the baseline WBC count was 2.48 (1.77, 2.86) × 10^9^/L, but these WBC counts did not show further reduction after AZA. Only one patient was *NUDT15* heterozygous (CT) but did not show progressive leukopenia after AZA therapy. Only one patient discontinued AZA because of gastrointestinal reactions; mycophenolate mofetil was subsequently administered. The maintenance dosage for the 13 patients with pre-therapy leukopenia was 1.13 (0.94, 1.60) mg ⋅ kg^−1^ ⋅ d^−1^) (Table 4).

**Table 4:** Subgroup analysis of outcome of patients with leukopenia at baseline.

Patient	Sex	WBC count at baseline	Decompensated cirrhosis	Hypersplenism	rs116855232 (C > T)	rs1142345 (T > C)	Progressive leukopenia	Follow-up (months)	Maintenance dosage (mg ⋅ kg^−1^ ⋅ d^−1^)	Azathioprine interruption	Interruption reason
1	F	2.99	No	No	CC	TT	No	17	1.87	No	–
2	F	2.91	No	Yes	CT	TT	No	16	1.14	No	–
3	F	2.80	No	No	CC	TT	No	10	–	Yes	Gastro-intestinal reactions
4	F	2.67	No	Yes	CC	TT	No	16	0.92	No	–
5	F	1.6	Yes	Yes	CC	TT	No	14	1.11	No	–
6	F	2.65	No	No	CC	TT	No	31	0.83	No	–
7	F	1.89	No	Yes	CC	TT	No	11	–	No	–
8	F	2.21	No	No	CC	TT	No	9	1.69	No	–
9	F	2.48	No	No	CC	TT	No	9	–	No	–
10	F	2.76	No	No	CC	TT	No	3	–	No	–
11	F	1.65	Yes	Yes	CC	TT	No	6	1.32	No	–
12	F	2.41	No	Yes	CC	TT	No	4	–	No	–
13	M	1.53	No	Yes	CC	TT	No	5	1.00	No	–

### rs116855232 Genotype and Biochemical Response

Among the 93 patients who reached biochemical remission, 75 (80.6%) were wild-type homozygotes, and 17 (18.3%) subjects were heterozygotes. No differences were observed in the percentage of patients who achieved biochemical remission (*P* = 0.361) or the median time to biochemical remission (*P* = 0.161; 3.0 vs. 4.0 months, respectively) between the genotypes.

## Discussion

Our results found that the rs116855232 but not the rs1142345 genotype was significantly associated with AZA-induced leukopenia and the maintenance dose in Chinese patients with AIH and related cirrhosis. Safety and efficacy can be maintained in most heterozygous genotype patients on AZA at lower doses of approximately 1 mg ⋅ kg^−1^ ⋅ d^−1^.

The gene encoding the TPMT enzyme is highly polymorphic, thus leading to varying levels of enzyme activity in different individuals. However, only approximately 20% of leukopenia cases can be explained by *TPMT* genetic deficiencies. A large subset of patients with the wild-type genotype develop leukopenia, implying that the utility of routine *TPMT* testing is limited and that other factors are responsible for the inter-individual variation in thiopurine sensitivity (Ban et al., 2008; Fangbin et al., 2012; Zhu and Cao, 2012; Lee et al., 2015). The present study showed a 1.7% frequency of the risk *TPMT* allele in the analyzed population, which is similar to that reported in other studies involving Chinese IBD patients. This *TPMT* polymorphism is rare in Asian populations, and the lowest frequencies have been observed in Chinese populations (∼0.9%) (Fangbin et al., 2012; Zhu and Cao, 2012; Zhu et al., 2016). This prevalence is significantly lower than the 10% prevalence reported in European populations (Collie-Duguid et al., 1999). Hence, the lack of significance observed for rs1142345 in the present study is likely due to its low prevalence in East Asian populations and the small sample size of our cohort.

NUDT15 is a member of the nudix hydrolase enzyme family, which primarily consists of pyrophosphohydrolases that act on nucleoside diphosphates linked to other moieties. The function of NUDT15 is to hydrolyze 8-oxo-dGTP and 8-oxo-dGDP to 8-oxodGMP, which reduces their cytotoxic effects (Carter et al., 2015). This finding has been replicated by multiple independent studies and has expanded to several other *NUDT15* SNPs, including rs147390019 (induces p.Arg139His) (Moriyama et al., 2016). Pharmacological analyses and cellular drug response studies have determined that NUDT15 activity is not only controlled by genetic variants but also by *NUDT15* expression levels in the crystal structure. According to protein stability analysis in our previous study, Arg139Cys substantially changes the NUDT15 crystal structure by breaking an α-helix in the active domain and reducing the stability of mutated NUDT15 (Yan et al., 2018).

Recent studies have found an *NUDT15* polymorphism that was significantly associated with thiopurine-induced leukopenia in East Asian ALL and IBD patients, suggesting its potential as a promising biomarker for leukopenia following the initiation of thiopurine therapy in Asian populations. In, Yang et al. (2014) were the first to report that the *NUDT15* allele frequencies were 10.4% in Korean IBD patients. Additionally, an immunochip-based 2-stage association study revealed that rs116855232 was strongly associated with thiopurine-induced early leukopenia (*OR* = 35.6; *P* combined=4.88 × 10^−94^). This variant showed a sensitivity and specificity of 89.4 and 93.2% in Korean IBD patients, respectively (Yang et al., 2014). Subsequently, an association study involving 142 Japanese IBD patients treated with thiopurines replicated the association between R139C and adverse event (Kakuta et al., 2016). The association between R139C and early (<8 weeks) leukopenia (WBC< 3000 mm^−3^) was replicated in our Japanese IBD cohort (*P* = 1.92 × 10^−16^, odds ratio = 28.4), although the association with late leukopenia (>8 weeks) was not confirmed. Another study involving 253 Chinese IBD patients found that the *NUDT15* variant was correlated with the early, middle (8–24 weeks) and late (>24 weeks) phases of leukopenia (*OR* = 15.67, 12.06, and 3.91, respectively; *P* = 2.13 × 10^−19^, 4.51 × 10^−9^ and 0.022, respectively) (Zhu et al., 2016). In our study, the rs116855232 allelic frequency was 9.4%, which is similar to data obtained from Chinese IBD patients (Zhu et al., 2016). The rs116855232 risk allele frequency is high in East Asian (10.4%) and Hispanic (7.1%) populations but rare in Caucasian (0.46%) populations (see Footnote) (Lek et al., 2016). Hence, despite differences according to ethnicity, detecting rs116855232 prior to thiopurine treatment is important, particularly in East Asian and Hispanic populations. Safety and efficacy can be maintained in most *NUDT15* heterozygous genotype patients on AZA at lower doses of approximately 1 mg ⋅ kg^−1^ ⋅ d^−1^, which is the basic therapeutic dosage for AIH. We propose that an adjusted AZA dosage should be considered for heterozygous genotype patients, in a disease-independent manner (Yang et al., 2015; Kakuta et al., 2016; Shah et al., 2017). We also found that patients with *NUDT15* CT genotypes had much lower ratios of AZA dose to 6-TGN concentration, implying that physicians could recommend dosing by calculating the ratio according to the *NUDT15* genotypes. This new therapeutic monitoring method may have more clinical value than blood 6-TGN concentration alone.

In addition, no study has addressed the issues of cirrhosis, previous leukopenia, and genotype heterozygosity together in AIH and related cirrhosis. It is an independent and first verification in Chinese AIH patients. It seems that in patients with liver disease fibrosis stage may influence AZA metabolism, yet our data implied that leukopenia was not associated with advanced fibrosis or splenomegaly, although Heneghan et al. (2006) found that advanced fibrosis predicts AZA toxicity in AIH patients. Moreover, subgroup analysis of the outcome for the 13 patients with previous leukopenia before therapy indicated that the common dose of AZA may be safe and effective for patients with wild-type *NUDT15* homozygotes. However, further prospective validation studies with larger sample sizes are warranted to provide more robust evidence for this subgroup.

Our results provide confirmation of the impact of rs116855232 on AZA-induced leukopenia and tolerance dose in AIH patients. However, *TPMT* remains the most widely accepted predictor of thiopurine-induced leukopenia to date; current guidelines, including the clinical practice guidelines for AIH by EASL and AASLD, recommend *TPMT* genotyping prior to the initiation of AZA treatment (Manns et al., 2010; European Association for the Study of the Liver, 2015). Because the *NUDT15* and *TPMT* variants can coexist, as we have previously reported (Yang et al., 2017), and methods for the detection are commercially available, *NUDT15* and *TPMT* genotyping are suggested to be routinely conducted in all patients who have a plan to use AZA medication.

The present study has some limitations. First, this investigation was a single-center cohort study, and the link between *NUDT15* gene polymorphisms and AZA related leukopenia in patients with AIH and related cirrhosis should be further analyzed in larger replication cohorts. However, AIH is a rare disease and very few centers focus on non-Caucasian AIH patients and follow a large number of these patients in a database, and we suppose that our AIH cohort gives a fairly good representation of overall *NUDT15* genotypes distribution in Chinese AIH population. A second limitation is the possibility that we may have missed some rare mutations in the *TPMT* and *NUDT15* genes, given that we were testing only the two most common mutations in Asians as a cost-effective practice using commercially available tests. As we have found that novel rare *NUDT15* variants could improve predictive sensitivity of thiopurine-induced leukopenia in children with ALL recently (Zhu et al., 2018), more variants need to be explored in the future. Third, given the experimental approach, the AZA metabolites were only detected in 61 (40.9%) patients.

## Conclusion

We found that a *NUDT15* polymorphism (rs116855232) was significantly associated with AZA-induced leukopenia in Chinese patients with AIH and related cirrhosis. For patients with the heterozygous variants, low-dose AZA (0.96 mg ⋅ kg^−1^ ⋅ d^−1^) is safe for maintaining remission and achieving efficacy comparable to that observed in wild-type patients. Even for patients with previous leukopenia or cirrhosis, AZA treatment may not be contraindicated if *NUDT15* genotypes permitted.

## Ethics Statement

This study was carried out in accordance with the recommendations of the ethical guidelines and approved by the Ethics Committee of the West China Hospital, Sichuan University (no. 2013221) with written informed consent from all subjects. All subjects gave written informed consent in accordance with the Declaration of Helsinki.

## Author Contributions

XF and DY wrote the manuscript. LY and HX designed the research. XF, DY, and RM performed the research. XF, DY, and RM analyzed the data.

## Conflict of Interest Statement

The authors declare that the research was conducted in the absence of any commercial or financial relationships that could be construed as a potential conflict of interest.

## Footnotes

1 http://exac.broadinstitute.org/

## References

[B1] Ban H. Andoh A. Tanaka A. Tsujikawa T. Sasaki M. Saito Y. (2008). Analysis of thiopurine S-methyltransferase genotypes in japanese patients with inflammatory bowel disease. *Intern. Med.* 47 1645–1648. 10.2169/internalmedicine.47.1268 18827410

[B2] Carter M. Jemth A. S. Hagenkort A. Page B. D. Gustafsson R. Griese J. J. (2015). Crystal structure, biochemical and cellular activities demonstrate separate functions of MTH1 and MTH2. *Nat. Commun.* 6:7871. 10.1038/ncomms8871 26238318 PMC4532830

[B3] Chazouilleres O. Wendum D. Serfaty L. Montembault S. Rosmorduc O. Poupon R. (1998). Primary biliary cirrhosis-autoimmune hepatitis overlap syndrome: clinical features and response to therapy. *Hepatology* 28 296–301. 10.1002/hep.510280203 9695990

[B4] Collie-Duguid E. S. Pritchard S. C. Powrie R. H. Sludden J. Collier D. A. Li T. (1999). The frequency and distribution of thiopurine methyltransferase alleles in caucasian and asian populations. *Pharmacogenetics* 9 37–42. 10.1097/00008571-199902000-00006 10208641

[B5] Connell W. R. Kamm M. A. Ritchie J. K. Lennard-Jones J. E. (1993). Bone marrow toxicity caused by azathioprine in inflammatory bowel disease: 27 years of experience. *Gut* 34 1081–1085. 10.1136/gut.34.8.1081 8174958 PMC1374358

[B6] Czaja A. J. Carpenter H. A. (2006). Thiopurine methyltransferase deficiency and azathioprine intolerance in autoimmune hepatitis. *Dig. Dis. Sci.* 51 968–975. 10.1007/s10620-006-9336-5 16773433

[B7] Dervieux T. Boulieu R. (1998). Simultaneous determination of 6- thioguanine and methyl 6-mercaptopurine nucleotides of azathioprine in redblood cells by HPLC. *Clin. Chem.* 44 551–555. 9510860

[B8] European Association for the Study of the Liver (2015). EASL clinical practice guidelines: autoimmune hepatitis. *J. Hepatol.* 63 971–1004. 10.1016/j.jhep.2015.06.030 26341719

[B9] Fangbin Z. Xiang G. Minhu C. Liang D. Feng X. Min H. (2012). Should thiopurine methyltransferase genotypes and phenotypes be measured before thiopurine therapy in patients with inflammatory bowel disease? *Ther. Drug. Monit.* 34 695–701. 10.1097/FTD.0b013e3182731925 23149442

[B10] Fei X. Shu Q. Zhu H. Hua B. Wang S. Guo L. (2018). NUDT15 r139c variants increase the risk of azathioprine-induced leukopenia in chinese autoimmune patients. *Front. Pharmacol.* 9:460. 10.3389/fphar.2018.00460 29867468 PMC5949564

[B11] Heneghan M. A. Allan M. L. Bornstein J. D. Muir A. J. Tendler D. A. (2006). Utility of thiopurine methyltransferase genotyping and phenotyping, and measurement of azathioprine metabolites in the management of patients with autoimmune hepatitis. *J. Hepatol.* 45 584–591. 10.1016/j.jhep.2006.05.011 16876902

[B12] Hindorf U. Jahed K. Bergquist A. Verbaan H. Prytz H. Wallerstedt S. (2010). Characterisation and utility of thiopurine methyltransferase and thiopurine metabolite measurements in autoimmune hepatitis. *J. Hepatol.* 52 106–111. 10.1016/j.jhep.2009.10.004 19906459

[B13] Hirschfield G. M. Beuers U. Corpechot C. Invernizzi P. Jones D. Marzioni M. (2017). EASL clinical practice guidelines: the diagnosis and management of patients with primary biliary cholangitis. *J. Hepatol.* 67 145–172. 10.1016/j.jhep.2017.03.022 28427765

[B14] Kakuta Y. Naito T. Onodera M. Kuroha M. Kimura T. Shiga H. (2016). NUDT15 R139C causes thiopurine-induced early severe hair loss and leukopenia in japanese patients with IBD. *Pharmacogenomics J.* 16 280–285. 10.1038/tpj.2015.43 26076924

[B15] Kim J. H. Cheon J. H. Hong S. S. Eun C. S. Byeon J. S. Hong S. Y. (2010). Influences of thiopurine methyltransferase genotype and activity on thiopurine-induced leukopenia in korean patients with inflammatory bowel disease: a retrospective cohort study. *J. Clin. Gastroenterol.* 44 e242–e248. 10.1097/MCG.0b013e3181d6baf5 20308917

[B16] Kim S. Y. Shin J. H. Park J. S. Kang S. Y. Nam T. S. Kim J. K. (2017). NUDT15 p.R139C variant is common and strongly associated with azathioprine-induced early leukopenia and severe alopecia in korean patients with various neurological diseases. *J. Neurol. Sci.* 378 64–68. 10.1016/j.jns.2017.04.041 28566182

[B17] Lee K. M. Kim Y. S. Seo G. S. Kim T. O. Yang S. K. Ibd Study Group of the Korean Association for the Study of Intestinal Diseases. (2015). Use of thiopurines in inflammatory bowel disease: a consensus statement by the korean association for the study of intestinal diseases (KASID). *Intest. Res.* 13 193–207. 10.5217/ir.2015.13.3.193 26130993 PMC4479733

[B18] Lee Y. J. Hwang E. H. Park J. H. Shin J. H. Kang B. Kim S. Y. (2016). NUDT15 variant is the most common variant associated with thiopurine-induced early leukopenia and alopecia in korean pediatric patients with crohn’s disease. *Eur. J. Gastroenterol. Hepatol.* 28 475–478. 10.1097/meg.0000000000000564 26735160

[B19] Lek M. Karczewski K. J. Minikel E. V. Samocha K. E. Banks E. Fennell T. (2016). Analysis of protein-coding genetic variation in 60,706 humans. *Nature* 536 285–291. 10.1038/nature19057 27535533 PMC5018207

[B20] Manns M. P. Czaja A. J. Gorham J. D. Krawitt E. L. Mieli-Vergani G. Vergani D. (2010). Diagnosis and management of autoimmune hepatitis. *Hepatology* 51 2193–2213. 10.1002/hep.23584 20513004

[B21] Manns M. P. Lohse A. W. Vergani D. (2015). Autoimmune hepatitis–Update 2015. *J. Hepatol.* 62 S100–S111. 10.1016/j.jhep.2015.03.005 25920079

[B22] Montano-Loza A. J. Czaja A. J. (2015). Cell mediators of autoimmune hepatitis and their therapeutic implications. *Dig. Dis. Sci.* 60 1528–1542. 10.1007/s10620-014-3473-z 25487192

[B23] Moriyama T. Nishii R. Perez-Andreu V. Yang W. Klussmann F. A. Zhao X. (2016). NUDT15 polymorphisms alter thiopurine metabolism and hematopoietic toxicity. *Nat. Genet.* 48 367–373. 10.1038/ng.3508 26878724 PMC5029084

[B24] Shah S. A. Paradkar M. Desai D. Ashavaid T. F. (2017). Nucleoside diphosphate-linked moiety X-type motif 15 C415T variant as a predictor for thiopurine-induced toxicity in Indian patients. *J. Gastroenterol. Hepatol.* 32 620–624. 10.1111/jgh.13494 27416873

[B25] Takatsu N. Matsui T. Murakami Y. Ishihara H. Hisabe T. Nagahama T. (2009). Adverse reactions to azathioprine cannot be predicted by thiopurine S-methyltransferase genotype in Japanese patients with inflammatory bowel disease. *J. Gastroenterol. Hepatol.* 24 1258–1264. 10.1111/j.1440-1746.2009.05917.x 19682195

[B26] Tanaka Y. Kato M. Hasegawa D. Urayama K. Y. Nakadate H. Kondoh K. (2015). Susceptibility to 6-MP toxicity conferred by a NUDT15 variant in Japanese children with acute lymphoblastic leukaemia. *Br. J. Haematol.* 171 109–115. 10.1111/bjh.13518 26033531

[B27] Yan W. Zhou Y. H. Wang L. Xiao J. Li W. (2018). NUDT15 polymorphism and severe azathioprine-induced myelosuppression in a chinese man with pemphigus vulgaris. *Br. J. Dermatol.* 178 e40–e41. 10.1111/bjd.15840 28733976

[B28] Yang J. J. Landier W. Yang W. Liu C. Hageman L. Cheng C. (2015). Inherited NUDT15 variant is a genetic determinant of mercaptopurine intolerance in children with acute lymphoblastic leukemia. *J. Clin. Oncol.* 33 1235–1242. 10.1200/jco.2014.59.4671 25624441 PMC4375304

[B29] Yang S. K. Hong M. Baek J. Choi H. Zhao W. Jung Y. (2014). A common missense variant in NUDT15 confers susceptibility to thiopurine-induced leukopenia. *Nat. Genet.* 46 1017–1020. 10.1038/ng.3060 25108385 PMC4999337

[B30] Yang X. Xu H. Yang J. Yang L. (2017). Rare gene variants in a patient with azathioprine-induced lethal myelosuppression. *Ann. Hematol.* 96 2131–2133. 10.1007/s00277-017-3112-9 28831540 PMC5691095

[B31] Yin D. Xia X. Zhang J. Zhang S. Liao F. Zhang G. (2017). Impact of NUDT15 polymorphisms on thiopurines-induced myelotoxicity and thiopurines tolerance dose. *Oncotarget* 8 13575–13585. 10.18632/oncotarget.14594 28088792 PMC5355121

[B32] Zhu Q. Cao Q. (2012). Thiopurine methyltransferase gene polymorphisms and activity in chinese patients with inflammatory bowel disease treated with azathioprine. *Chin. Med. J.* 125 3665–3670. 23075721

[B33] Zhu X. Wang X. D. Chao K. Zhi M. Zheng H. Ruan H. L. (2016). NUDT15 polymorphisms are better than thiopurine S-methyltransferase as predictor of risk for thiopurine-induced leukopenia in chinese patients with crohn’s disease. *Aliment. Pharmacol. Ther.* 44 967–975. 10.1111/apt.13796 27604507

[B34] Zhu Y. Yin D. Su Y. Xia X. Moriyama T. Nishii R. (2018). Combination of common and novel rare NUDT15 variants improves predictive sensitivity of thiopurine-induced leukopenia in children with acute lymphoblastic leukemia. *Haematologica* 103 e293–e295. 10.3324/haematol.2018.187658 29519865 PMC6029522

