# Supplementary material for: NUDT15 Polymorphism Confer Increased Susceptibility to Thiopurine-Induced Leukopenia in Patients With Autoimmune Hepatitis and Related Cirrhosis
Source: Front Pharmacol. 2019 Apr 9;10:346. doi: 10.3389/fphar.2019.00346 (PMC6465603; doi:10.3389/fphar.2019.00346)
Supplement: Supplementary file 1 [file Table_1.doc]

**Supplement Table S1 6-TGN concentration in different characteristics**

| Characteristics | 6-TGN  (pmol/8*10^8^RBC) | *P* |
| --- | --- | --- |
| Gender |  | 0.379 |
| female | 152.9(95.0, 233.9) |  |
| male | 98.2(90.8, 233.2) |  |
| AZA maintenance dosage |  | 0.637 |
| ≤1.0mg^.^kg^-1.^d^-1^ | 144.9(123.3, 300.2) |  |
| >1.0mg^.^kg^-1.^d^-1^ | 183.8(90.8, 235.3) |  |
| Leukopenia |  | 0.149 |
| With leukopenia | 198.8(145.1, 857.9) |  |
| Without leukopenia | 149.1(91.1, 231.3) |  |
| Severity |  | 0.757 |
| With cirrhosis | 153.9(90.0, 247.7) |  |
| Without cirrhosis | 149.1(94.4, 217.3) |  |
| rs116855232 |  | 0.002 |
| CC | 145.3(87.4, 199.0) |  |
| CT | 276.6(150.9, 501.0) |  |
| rs1142345 |  | - |
| TT | 149.0(93.0, 231.3) |  |
| TC | 430.8 |  |
